# The Role of Neuroinflammation in Shaping Neuroplasticity and Recovery Outcomes Following Traumatic Brain Injury: A Systematic Review

**DOI:** 10.3390/ijms252111708

**Published:** 2024-10-31

**Authors:** Andrea Calderone, Desirèe Latella, Davide Cardile, Antonio Gangemi, Francesco Corallo, Carmela Rifici, Angelo Quartarone, Rocco Salvatore Calabrò

**Affiliations:** 1 Department of Clinical and Experimental Medicine, University of Messina, Piazza Pugliatti 1, 98122 Messina, Italy; 2 IRCCS Centro Neurolesi Bonino-Pulejo, S.S. 113 Via Palermo, C.da Casazza, 98124 Messina, Italy; desiree.latella@irccsme.it (D.L.); davide.cardile@irccsme.it (D.C.); antonio.gangemi@irccsme.it (A.G.); francesco.corallo@irccsme.it (F.C.); carmela.rifici@irccsme.it (C.R.); angelo.quartarone@irccsme.it (A.Q.); roccos.calabro@irccsme.it (R.S.C.)

**Keywords:** traumatic brain injury, neuroinflammation, neuroplasticity, neurorehabilitation

## Abstract

Neuroplasticity and neuroinflammation are variables seen during recovery from traumatic brain injury (TBI), while biomarkers are useful in monitoring injury and guiding rehabilitation efforts. This systematic review examines how neuroinflammation affects neuroplasticity and recovery following TBI in animal models and humans. Studies were identified from an online search of the PubMed, Web of Science, and Embase databases without any search time range. This review has been registered on Open OSF (n) UDWQM. Recent studies highlight the critical role of biomarkers like serum amyloid A1 (SAA1) and Toll-like receptor 4 (TLR4) in predicting TBI patients’ injury severity and recovery outcomes, offering the potential for personalized treatment and improved neurorehabilitation strategies. Additionally, insights from animal studies reveal how neuroinflammation affects recovery, emphasizing targets such as NOD-like receptor family pyrin domain-containing 3 (NLRP3) and microglia for enhancing therapeutic interventions. This review emphasizes the central role of neuroinflammation in TBI, and its adverse impact on neuroplasticity and recovery, and suggests that targeted anti-inflammatory treatments and biomarker-based personalized approaches hold the key to improvement. Such approaches will need further development in future research by integrating neuromodulation and pharmacological interventions, along with biomarker validation, to optimize management in TBI.

## 1. Introduction

**Figure 1:**
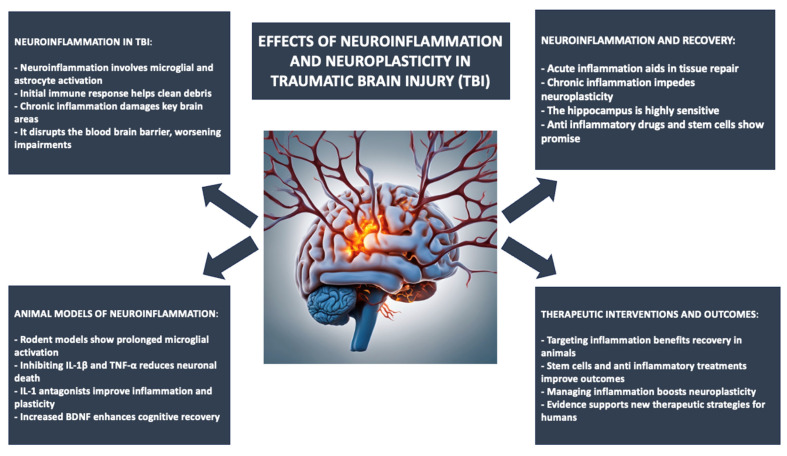
The effects of neuroinflammation and neuroplasticity in TBI patients and animals.

Brain injuries caused by external forces can lead to a variety of cognitive, physical, and psychosocial issues, resulting in a complex condition known as TBI [1,2,3,4,5,6]. It is categorized as mild, moderate, or severe depending on symptoms and diagnostic imaging results. TBI is one of the main reasons for disability and mortality, especially in young adults and older individuals, with males being the most impacted because of falls, traffic accidents, and sports injuries [7,8,9,10,11]. Signs may involve head pain, disorientation, as well as enduring complications such as memory difficulties and alterations in personality [12,13,14,15]. The Glasgow Coma Scale (GCS) is utilized for evaluating the seriousness of TBI, with a scale from 3 to 15 to show different levels of neurological functionality [16,17,18,19]. Damage to the brain caused by TBI demonstrates the brain’s impressive ability to reorganize and adapt, known as neuroplasticity. Undamaged neurons near the injury can form fresh connections, leading to some improvement in functionality [20,21]. Rehabilitation methods, such as cognitive therapy, motor imagery, and virtual reality interventions, can increase neuroplasticity by activating the damaged parts of the brain [22]. Nonetheless, severe injuries or neuroinflammation can interrupt these functions, leading to enduring deficits in cognitive abilities, motor skills, and emotional control. The balance between brain damage, neuroinflammation processes, and the neural network reorganization ability is at the core of clinical outcomes after TBI [23,24,25,26,27,28,29,30,31,32,33,34]. In TBI, neuroinflammation can be identified by the brain’s immune reaction to trauma, involving the stimulation of microglia and astrocytes, the secretion of pro-inflammatory cytokines, and heightened oxidative stress [35,36]. The first immune reaction is defensive; it helps to remove waste and encourages the healing process. Yet, persistent neuroinflammation becoming a long-term issue stimulates harm to neurons, resulting in additional injury [37,38]. The most crucial areas affected by neuroinflammation after TBI are the hippocampus, cortex, and white matter tracts, impacting cognitive memory and motor function. Primarily, it is recognized as a key contributor to these processes, capable of releasing inflammatory agents like tumor necrosis factor-alpha (TNF-α), interleukin-1 beta (IL-1β), and interleukin-6 (IL-6), known to trigger pathways resulting in cell death and harming the blood–brain barrier [39,40]. Interference with the latter enables immune cells from outside to access the brain, causing higher levels of inflammation and negative effects on cognitive abilities and overall function even once the injury has healed [41,42]. Moreover, neuroinflammation could lead to synaptic dysfunction, causing the brain to lose its ability to effectively communicate between neural networks [43,44]. This ongoing inflammation is believed to be the primary cause of chronic traumatic encephalopathy and the development of other neurodegenerative diseases in individuals who have experienced a TBI in the past [45,46,47,48,49,50,51,52,53,54,55]. While neuroinflammation can be damaging, it also plays a role in tissue healing and supports the growth of new nerve cells. During the initial phase following an injury, inflammation is needed to clear away dead cells and debris while also creating an environment for future tissue repair and nerve growth. On the other hand, alternative approaches such as giving anti-inflammatory medications or using stem cell treatment to control inflammation have shown potential in enhancing recovery and flexibility [56,57,58]. These interventions can help lessen the negative effects of long-lasting inflammation without harming the positive aspects of inflammation, leading to improved functional results following TBI [59,60,61,62]. Animal studies have thus far been quite instructive regarding the mechanisms of neuroinflammation and the impact on neuroplasticity following TBI. Rodent models of TBI show microglial activation that starts within hours following injury, is sustained for weeks, and thereby both acutely and chronically contributes to damage [63,64]. Animal models have demonstrated that the inhibition of specific inflammatory pathways, such as either IL-1β or TNF-α signaling, leads to striking reductions in neuronal death and improvement in cognitive outcomes following injury [65,66]. Indeed, interleukin-1 (IL-1) receptor antagonists have been given in models of animal injury that exhibit less inflammation and increased synaptic plasticity in the hippocampus [67]. Furthermore, animal models demonstrate that neuroinflammation induced by TBI severely impairs the production of brain-derived neurotrophic factor (BDNF), which is an important molecule favoring neurogenesis and synaptic plasticity [68,69]. In all those models in which the expression of BDNF has been maintained or increased, cognitive recovery has been substantially improved [70,71]. More recently, stem cells have been widely employed in animal models of TBI. These, once combined with anti-inflammatory treatments, have represented tissue repair and functional recovery that is significantly enhanced [72]. The need for precision has necessitated the employment of monitoring tools such as biomarkers, which are important in the assessment of injury, neuroinflammation, and the effectiveness of rehabilitation. Biomarkers present in blood, cerebrospinal fluid, or detected through imaging are essential for evaluating damage and rehabilitation in patients with TBI. They offer clinicians information on the seriousness of brain damage and help in tracking neuroinflammation and neuroplasticity [73,74]. Most biomarkers are crucial for swift TBI detection and outcome prediction. Prominent instances include S100 Calcium-binding Protein B (S100B) and Glial Fibrillary Acidic Protein (GFAP), which suggest neuronal damage and astrocyte activation [75,76,77]. Increased levels of these proteins are linked to worse results, enabling interventions to be made promptly [78]. Detecting biomarkers early is associated with better rehabilitation results. Moreover, neuroimaging biomarkers, such as magnetic resonance imaging (MRI) and positron emission tomography (PET), demonstrate changes in the structure and function of the brain because of therapies like cognitive training over time [79,80,81]. Biomarkers such as S100B and GFAP can also reflect the levels of neuroinflammation, with elevated levels indicating significant inflammation that hinders the healing process [82,83,84]. The joint use of neuroinflammation and neuroplasticity markers improves the comprehension of patient recovery patterns, indicating effective treatment and positive progress when inflammatory markers decrease and neuroplasticity markers such as BDNF increase [85,86]. This two-pronged strategy can improve treatment plans by reducing damaging inflammation and promoting positive changes in brain flexibility [87,88]. The present systematic review summarizes evidence related to neuroinflammation that modulates neuroplasticity and recovery both in animal models and humans after TBI and draws some implications for neurorehabilitation. Such insights will allow public health and clinical practice to develop targeted, specific interventions aimed at modulating neuroinflammation and possibly improving the outcomes of patients with TBI. For a better understanding of the topic, we provided a summary of the effects of neuroinflammation and neuroplasticity in TBI patients and animals in Figure 1.

## 2. Methods

### 2.1. Search Strategy

**Figure 2:**
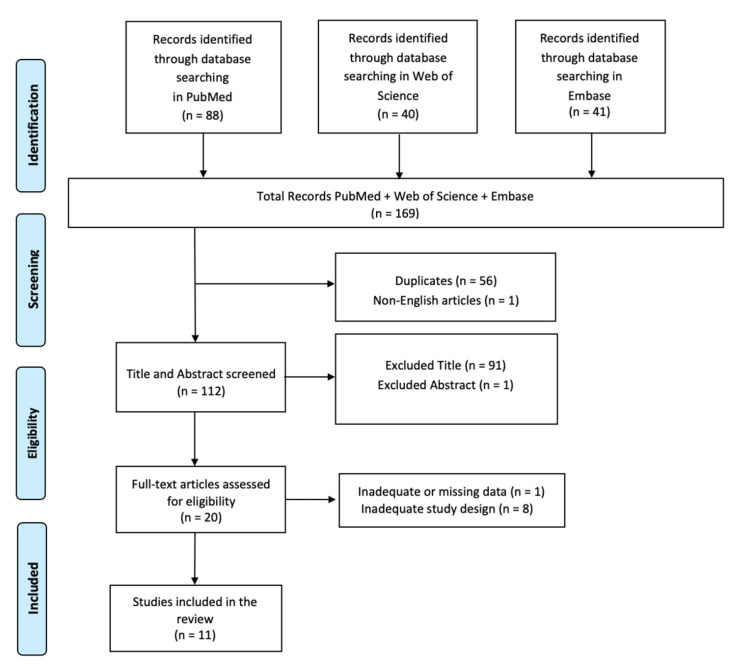
PRISMA 2020 flow diagram of evaluated studies.

A comprehensive literature search was performed using the PubMed, Web of Science, and Embase databases, employing the keywords: (All Fields: “Traumatic Brain Injury”) AND (All Fields: “Neuroinflammation”) AND (All Fields: “Neuroplasticity”) without any search time range. The PRISMA (Preferred Reporting Items for Systematic Reviews and Meta-Analyses) flow diagram was utilized to outline the process (identification, screening, eligibility, and inclusion) for selecting relevant studies, as illustrated in Figure 2. Titles and abstracts from the database searches were independently reviewed. Articles were evaluated for their relevance based on predefined inclusion criteria. All titles and abstracts that met these criteria were fully reviewed. Multiple expert teams independently selected articles and analyzed data to minimize bias, discussing discrepancies until a consensus was achieved. This review has been registered on Open OSF (n) UDWQM.

### 2.2. PICO Evaluation

We applied the PICO model (Population, Intervention, Comparison, Outcome) to create our search terms.

The population involved includes animal models and humans who have TBI. The intervention is the impact of neuroinflammation on neuroplasticity and recovery processes. The comparison refers to no/minimal/controlled neuroinflammation against higher levels of neuroinflammation. The primary outcome of this review will involve an analysis of how different levels of neuroinflammation affect neuroplasticity and, ultimately, recovery outcomes, offering insight into potential therapeutic strategies aimed at enhancing recovery after TBI.

### 2.3. Inclusion Criteria

A study was included if it described or examined how neuroinflammation affects neuroplasticity and recovery following TBI in animal models and humans. Only articles written in English were considered. Additionally, studies that described or investigated the functional assessment of these patients were included. We only included studies conducted in human populations and published in English that met the following criteria: (i) original or protocol studies of any kind; and (ii) articles that examine how neuroinflammation affects neuroplasticity and recovery following TBI in animals and humans.

### 2.4. Exclusion Criteria

A study was excluded if it lacked data or information regarding how neuroinflammation affects neuroplasticity and recovery following TBI in animal models and humans. Systematic, integrated, or narrative reviews were also excluded; however, their reference lists were reviewed and included when relevant. Additionally, any articles written in languages other than English were excluded.

## 3. Results and Discussion

### 3.1. Quality of Included Studies—Risk of Bias

**Figure 3:**
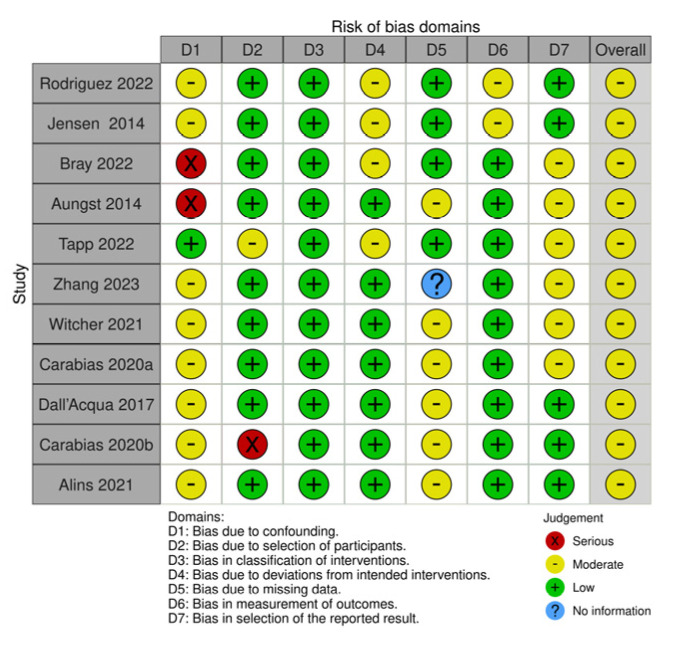
The Cochrane risk of bias in non-randomized studies of interventions (ROBINS-I) [89,90,91,92,93,94,95,96,97,98,99].

We assessed the risk of bias using appropriate tools based on the design of the included studies. For all non-randomized studies [89,90,91,92,93,94,95,96,97,98,99], including eight experimental studies [89,90,91,92,93,94,95,99], two cohort studies [96,97], and one prospective observational study [98], we applied the ROBINS-I tool. ROBINS-I assesses bias in seven areas: (i) bias due to confounding, (ii) bias in participant selection, (iii) bias in classification of interventions, (iv) bias due to deviations from intended interventions, (v) bias due to missing data, (vi) bias in outcome measurement, and (vii) bias in selection of the reported outcome (Figure 3) [100].

The ROBINS-I ratings of the eleven studies indicate good methodological quality, although each study has notable areas of concern. All studies except Tapp et al. [93] presented some issues with the D1 domain (bias due to confounding), with a serious risk in the Bray et al. [91] and Aungst et al. [92] studies. Tapp et al. [93] and Carabias et al. [96] reported, respectively, a moderate and a serious risk in the selection of participants (D2), while all others showed a low risk in this domain. The classification of interventions (D3) was made appropriately in all the analyzed research, demonstrating a low risk, while a moderate risk regarding the deviations from intended interventions (D4) was detected in Rodriguez et al. [89], Jensen et al. [90], Bray et al. [91], and Tapp et al. [93]. There was no information about bias due to missing data (D5) in the Zhang et al. [94] study, while a moderate risk was displayed in the outcome measure (D6) in Rodriguez et al. [89] and Jensen et al. [90]. Rodriguez et al. [89], Jensen et al. [90], Dell’Acqua et al. [97], Carabias et al. [98], and Alins et al. [99] demonstrated low risk in the selection of the reported result (D7), while all others presented moderate risk in this domain. Overall, while most studies showed a low risk of bias in several domains, specific areas such as confounding variables, missing data, and the selection of reported outcomes remain critical points to examine to ensure the reliability and validity of the study results.

### 3.2. Synthesis of Evidence

In total, 169 articles were found: 56 articles were removed due to duplication after screening; 1 article was excluded because it was not published in English; and 92 articles were excluded based on title and abstract screening. Finally, 9 articles were removed based on screening for inadequate and untraceable study designs (Figure 2). Therefore, eleven research articles met the inclusion criteria and were included in the review. These studies are summarized in Table 1.

The studies discussed in this review examine how neuroinflammation affects neuroplasticity and recovery following TBI in animal models and humans. Seven articles analyzed the mechanisms of neuroinflammation and recovery from different animal studies [89,90,91,92,93,94,95]; the other four papers investigated the role of biomarkers in predicting injury severity and recovery in humans [96,97,98,99].

### 3.3. Neuroinflammation and Recovery Mechanisms in TBI: Insights from Recent Animal Studies

Recent studies have used a range of biomarkers, cellular mechanisms, and treatment options to explore the complex interactions of neuroinflammation with recovery after TBI. The following series of articles considers how different factors, from molecular pathways to disturbed sleep, affect the severity of the injury, the pattern of recovery, and eventual outcomes. In an experimental study on animals [89], the NLRP3 inflammasome was investigated as the critical regulator of neuroinflammation after TBI using a closed-head injury model. Transcriptional and behavioral profiles were examined at 24 h post-TBI using both pharmacological approaches with MCC950 at 3 mg/kg pre-/post-injury and genetic approaches in NLRP3 knockout mice. For example, wild-type mice developed a robust pro-inflammatory response characterized by highly expressed inflammasome components, microglia, astrocyte markers, and cytokines. Interleukin-1 beta (IL-1β, an inflammatory cytokine) production did not change in the NLRP3 knockout or wild-type mice, very surprisingly. No compensatory activation of other inflammasomes occurred. The overexpression of several markers for microglia and astrocytes by the knockout mice explained an increased cytokine response. These findings highlight the transient yet critical role of NLRP3 activation in modulating the glial cell response and neuroinflammation after TBI [89]. Jensen et al. [90] investigated the leukotrienes, inflammatory lipid mediators involved in secondary brain damage following TBI. In normal physiological conditions, these leukotrienes are not detectable in the brain, but are rapidly synthesized following trauma as an outcome of an interaction between neutrophils and brain cells. Testing was performed regarding the efficacy of a 5-lipoxygenase-activating protein (FLAP) inhibitor-MK-886 against leukotriene production, brain damage, synaptic dysfunction, and cognitive impairment. Male Sprague–Dawley rats with moderate unilateral fluid percussion injury were administered MK-886 either before or right after the injury occurred. Treatment with MK-886 potently inhibited leukotriene synthesis, reduced brain edema, and minimized the extent of blood–brain barrier disruption in the CA1 hippocampus. It also improved long-term potentiation as well as performance in spatial learning and memory. These results support the concept that leukotrienes represent major contributors to secondary brain injury and cognitive deficits. It, therefore, follows that FLAP inhibitors, including MK-886, are an especially promising anti-inflammatory intervention strategy aimed at the prevention or treatment of brain injury and related cognitive deficits induced by TBI, possibly also improving neurorehabilitation [90]. Another paper investigated whether microglial replacement delays chronic neuroinflammation and accelerates recovery after TBI. The brain immune cells, microglial cells, have been reported to become hyper-reactive following TBI, leading to chronic inflammation and subsequent neurodegenerative complications. Along this line, the authors induced microglial turnover on the seventh day following midline fluid percussion injury with a colony-stimulating factor 1 receptor antagonist known as PLX5622 in male mice. After 30 days following the injury, microglial turnover prevented 90% of TBI-related gene expression changes in the cortex linked to neuropathology. This intervention also reduced neuronal connectivity deficits after 30 days post-injury. However, changes in dendrites and myelin persisted. Despite this fact, microglial turnover significantly improved depressive-like behaviors and cognitive deficits. Moreover, microglial turnover decreased the heightened immune reaction following the lipopolysaccharide challenge. These findings indicate that forced microglial turnover reduces chronic inflammation and improves behavioral and cognitive outcomes after TBI [91]. Another experimental work presented the chronic effects of repeated, mild traumatic brain injury (mTBI) induced by a rat lateral fluid percussion model on neuronal health, neuroinflammation, and cognitive function. At post-injury day 28, multiple mTBI led to notable cell loss of neurons and higher levels of activated microglia in both the hippocampus on the same side as the injury and the opposite side. The electrophysiological recordings showed that long-term potentiation, a measure of synaptic plasticity, cannot be induced in hippocampal slices in either hemisphere following repeated mTBI. While the field excitatory postsynaptic potential responses mediated via the NMDA receptor were significantly depressed, those mediated via the AMPA receptor remained intact. The induction of long-term potentiation was unhindered from the single mTBI; potentiation was robust in the ipsilateral hippocampus. Repeated mTBI thus resulted in severe cognitive dysfunction, as evidenced by a dismal performance in both the Morris water maze and novel object recognition tests. Taken together, data suggest that repeated mTBI disrupts synaptic neurotransmission and therefore impairs hippocampal function in addition to chronic neuroinflammation and neurodegeneration [92]. Tapp and colleagues examined the impact of sleep fragmentation (SF), a frequent sequela of TBI, on recovery, postulating that stress-associated sleep fragmentation involves abnormal activation of the hypothalamic–pituitary–adrenal (HPA) axis and exacerbates neuroinflammation, promoting poorer recovery outcomes. Male and female mice with moderate TBI either remained undisturbed or were subjected to daily SF starting either 7 or 30 days post-injury. The results indicated that SF enhanced cortical expression of stress-related genes and depressed the transcriptional activity of glucocorticoid receptor (GR), a major regulator of stress response. SF also enhanced microglial activation and pro-inflammatory signaling in both the cortex and hippocampus, accompanying depressed hippocampal neuronal activity and defective HPA-axis responses. At 30 days post-injury, SF caused additional suppression of hippocampal function and defective cognitive performance, as determined by trace fear conditioning. Hippocampal infusions with a GR agonist also restored GR activity in TBI mice. This finding underlines the role of GR dysregulation in post-injury stress responses. Altogether, this evidence suggests that SF acts through the engagement of HPA-axis dysfunction after TBI to drive neuroinflammation and blunted cognitive recovery [93]. Zhang et al. discussed the roles that microRNAs, present in mesenchymal stem cell (MSC)–small extracellular vesicles (sEVs), could play in promoting recovery after TBI. The researchers investigated the therapeutic efficacy of sEVs derived from naïve MSCs and MSCs overexpressing a scramble control shRNA (short hairpin RNA is a type of RNA molecule with a tight hairpin turn that can be used to silence gene expression), and MSCs knocked down Argonaute 2, a protein essential to the miRNA machinery. Animals were intravenously infused with these sEVs or placebo one day after injury. Results indicated that sEVs from naïve and control-treated MSCs significantly improved neurological outcomes, reduced hippocampal neuronal loss, inhibited neuroinflammation, and enhanced neurovascular remodeling, including both angiogenesis and neurogenesis, whereas sEVs from Argonaute 2 knockdown MSCs with reduced miRNA content conferred only marginal benefits comparable to placebo treatment. Overall, these findings showed that the miRNAs of MSC-derived sEVs played an essential role in the recovery process, allowing for neuronal protection, reduction in inflammation, and, therefore, enhanced repair within the brain [94]. A final animal study investigated whether microglia involvement was related to acute, subacute, and chronic neuropathological developments after TBI. The authors administered PLX5622, a CSF1R antagonist that led to microglia depletion in male mice and followed neuropathology and cortical inflammation. Thus, inflammatory and neuropathological gene expression was generally independent of microglia and peaked early, at 1 day after injury. However, microglia depletion at 7 and 30 days after injury reversed most TBI-induced inflammatory and neurodegenerative gene expressions, especially those associated with interferon signaling. Single-cell RNA sequencing showed that microglia depletion reversed neuronal suppression of genes critical for cognitive function, which were associated with dopamine signaling, synaptogenesis, and calcium signaling. Microglia depletion also prevented reductions in cortical dendritic complexity, neuronal connectivity, and cognitive deficits at 30 days. These findings further highlight the role of microglia in chronic neuroinflammation and functional deficits after TBI [95].

### 3.4. Advances in Neurorehabilitation: The Role of Biomarkers in Predicting Injury Severity and Recovery

**Figure 4:**
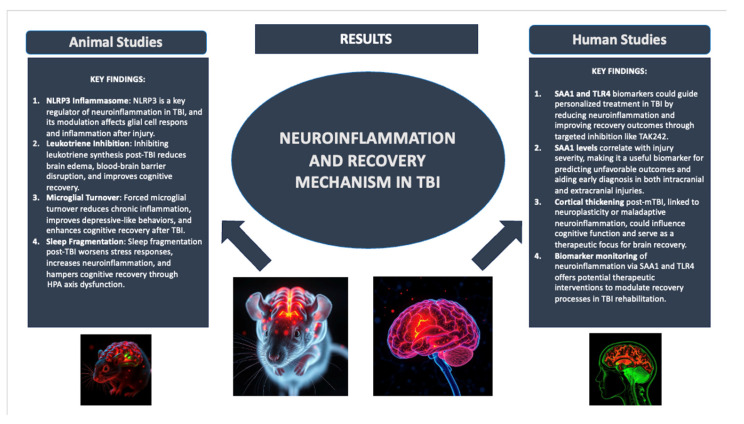
Neuroinflammation and recovery mechanism in TBI.

These papers emphasize the important function of biomarkers in the neurorehabilitation plan for TBI patients. They show not only how specific biomarkers like SAA1 and TLR4 are associated with the severity of injury and recovery outcome, but also how these biomarkers could guide personalized treatment and enhance the monitoring and management of patients. A prospective observation cohort study aimed to investigate the association between traumatic intracranial hemorrhage and serum biomarkers concerning the severity, volume, and location of traumatic intracranial hemorrhage in a series of 115 TBI patients. Biomarkers presented here included S100β, YKL-40, SAA1, procalcitonin, and C-reactive protein, whose intracranial hematoma volume correlation was measured with computed tomography. It would also indicate that biomarkers SAA1, YKL-40, PCT, and S100β are most closely associated with the overall GCS scores and motor scores, each reflecting the severity of brain injury. These biomarkers significantly shared a relationship with a variety of hemorrhages, namely subdural hematoma, subarachnoid hemorrhage, and intraparenchymal hemorrhage. The combination of some biomarkers increased the possibility of detecting intracranial bleeding and, therefore, for early diagnosis analysis [96]. A longitudinal cohort study investigated gray matter morphology in individuals with mTBI in the first year following injury and, importantly, examined the changes in brain structure over time between the patients and healthy controls. A total of 49 individuals with mTBI and 49 without injury underwent high-resolution MRI imaging within 7 days and 1 year post-injury. This study examined the thickness of the cortex, specifically in the frontal, parietal, and temporal regions of the brain. These analyses revealed a significant group-by-time interaction in the prefrontal cortex: whereas controls showed normal cortical thinning, mTBI patients showed cortical thickening. In patients with positive results, there was a correlation between slight cortical thickening and improved cognitive function, while excessive cortical thickening in patients with negative results was associated with declining performance. This would suggest that structural changes across the prefrontal cortex after mTBI reflect a favorable neuroplasticity or a maladaptive neuroinflammation, at least in part [97]. Another prospective observational study focuses on the finding of novel peptide biomarkers for intracranial and extracranial injuries in TBI patients and their outcomes. Serum samples obtained from 20 TBI patients were analyzed by mass spectrometry to discover biomarkers. Thereafter, one SAA1 was identified as a putative biomarker that best reflects both intracranial and extracranial injury severity. High levels of SAA1 are closely associated with markers of intracranial lesion volume, clinical severity, and systemic secondary insults. The SAA1 levels were also associated with traditional injury markers including S100β, GFAP, NSE, total tau (T-tau), and pNF-H. SAA1 further predicted an unfavorable outcome and mortality with high accuracy both at hospital discharge and 6 months later [98]. In a final study, the SAA1 protein was studied along with its interaction with TLR4, which was found to be correlated with serum involved in TBI and mRNA obtained from white blood cells of patients who suffered from TBI. The results indicated that higher SAA1 levels correlated with injury severity and 6-month patient outcomes. Additionally, a relationship was observed between the concentrations of SAA1 and the existence of TLR4 mRNA in leukocytes. Cell culture experiments showed that SAA1 facilitated the feed-forward expression of TLR4, which enhanced the release of inflammatory cytokines and further up-regulated SAA1 levels. Furthermore, intraperitoneally administered TAK242, a TLR4 antagonist, was found to reduce SAA1 levels, improve neurobehavioral functions, and prevent blood–brain barrier disruption in TBI models. The implications for neurorehabilitation are great. Specific inhibitors of the SAA1-TLR4 axis, such as TAK242, can be exploited to reduce neuroinflammation and may therefore support the recovery process in TBI patients. This further highlights that SAA1 and TLR4 may serve as useful biomarkers and therapeutic targets in the treatment of TBI to help improve patient recovery outcomes [99]. A graphical abstract of the neuroinflammation and recovery mechanism is visualized in Figure 4.

### 3.5. Discussion

This systematic review explored how neuroinflammation affects neuroplasticity and recovery following TBI in animal models and humans. The reviewed articles indicated the critical use of neuroinflammation and biomarkers in TBI and recovery. Animal studies demonstrated that neuroinflammation, with defined determinants including the NLRP3 inflammasome and leukotrienes, persists at the core of TBI pathology. Chronic inflammation, caused by the NLRP3 inflammasome or leukotrienes, can suppress neuroplasticity with continuous neuronal injury and block the brain’s repair mechanisms. This would mean that targeting inflammation is one of the important strategies for encouraging recovery. Similarly, the interventions that may control microglia activity, their turnover, or the use of inhibitors are suggested by the research to improve neuroplasticity by reducing injurious inflammation and enhancing repair within the brain [89,90,91,92,93,94,95]. Biomarker evidence has underscored the importance of markers such as SAA1 and TLR4 in predicting injury severity and recovery. These biomarkers are important not only to assess the extent of brain damage, but also to guide therapeutic approaches in a personalized manner. Indeed, for example, SAA1 is closely related to injury severity and recovery outcome; the interaction of SAA1 with TLR4 presents a promising target to reduce neuroinflammation [96,97,98,99]. The information in the literature and the findings in this review enable us to make a significant observation in neurorehabilitation, specifically focusing on neuromodulation methods. Such techniques can be applied and used to restore both cognitive and motor functions in patients with TBI [101,102]. We have discussed how chronic neuroinflammation in TBI impairs neuroplasticity and inhibits the self-healing properties of the brain [103,104]. In this respect, neuromodulation techniques, namely transcranial magnetic stimulation (TMS) and transcranial direct current stimulation (tDCS), represent an interesting avenue of research, potentially offering improved outcomes of recovery by acting on some basic levels of the mechanisms of inflammation and enhancing neuroplasticity [105,106]. Of these many insights provided by animal models, neuroinflammation centrally mediated via the NLRP3 inflammasome and leukotriene in chronic inflammation following TBI would, perhaps, be the most important. Such chronic inflammation may interfere with synaptic plasticity, especially within highly restricted brain regions such as the hippocampus, which is thought to be involved in learning and memory and, thus, in general cognitive functioning [107,108]. In this regard, neuromodulation of those specific regions could be strategically used to regain neural connectivity and improve synaptic plasticity [109,110]. Stimulation of neural circuits compromised by chronic inflammation via TMS could act on improved long-term potentiation and synaptic efficiency towards further cognitive recovery [111,112]. Similarly, we have also seen that microglial activity is considered a major cause of the development of neuroinflammation and long-term failures in cognitive and behavioral functions [113,114]. Induction of microglial turnover in animal studies provided improved neuronal connectivity with lower inflammatory gene expression and improved cognition [115,116]. Therefore, it can be inferred that neuromodulation can also control microglia activity, reducing the hyperreactive state of these cells and the associated chronic inflammation [117,118]. tDCS may provide a noninvasive method by which modulation of cortical excitability and a reduction in inflammation could impact microglia behavior and improve neuroplasticity [119,120]. Biomarkers such as SAA1 and TLR4 are also important for personalization in neuromodulation strategies [121,122]. High levels of SAA1, indicative of severe neuroinflammation, result in poorer recovery outcomes and could guide clinicians in personalizing neuromodulation protocols. For example, it is possible that individuals with higher levels of SAA1 would benefit from more aggressive or targeted neuromodulation to address inflammation-related impairments [123]. Second, and additionally, the interaction between SAA1 and TLR4, which increases inflammation, is an important target for combining neuromodulation with pharmacological interventions [124]. TLR4 inhibitors such as TAK242 could, for example, be administered in a combination treatment with neuromodulation, leading to less inflammation and improving overall recovery [125,126]. It is crucial to comprehend the bio-physiology of the person with TBI when creating successful recovery plans. This is the location where specific biomarkers like SAA1 and TLR4 are identified and analyzed to gain important understandings about the severity of injury and possible path to recovery. By linking these biomarkers to clinical results, healthcare providers can customize rehabilitation plans to meet the specific requirements of each patient, ultimately improving the overall supervision of their recovery journey. For instance, the levels of certain biomarkers indicate the severity of intracranial injuries and could potentially guide healthcare providers in determining treatment plans and interventions, as we have seen. This knowledge can assist in directing timely therapeutic interventions that are essential for enhancing recovery results. The changing levels of biomarkers over time can also indicate shifts in the brain, such as neuroplasticity or harmful neuroinflammation. These patterns are important in distinguishing between a patient in good recovery and one experiencing injury complications. The importance of addressing neuroinflammation in recovery after TBI is highlighted by the relationship between biomarkers like SAA1 and inflammatory pathways. To achieve this goal, certain medications that target these pathways can reduce the inflammatory responses and minimize their negative impact on the healing process. This method aids in both short-term recovery and long-term functional enhancements. Ultimately, incorporating biomarker analysis into rehabilitation protocols revolutionizes the management of TBI, enabling a more proactive and personalized approach to patient care. Healthcare providers could improve recovery paths, enhance functional outcomes, and ultimately enhance the quality of life in individuals recuperating from TBI with this understanding [127,128,129,130,131,132].

The review has several strengths. Among the main ones is a very comprehensive and systematic literature search across multiple databases that ensured that relevant studies were covered. Adhering to the PRISMA guidelines enhances the transparency and reproducibility of the review process. In addition, a risk of bias analysis was performed on the eleven studies selected to increase the reliability of the results. The systematic approach provides a structured review of neuroinflammation concerning neuroplasticity and recovery in detail, with an integrated view from both animal and human studies. Furthermore, biomarkers are of value for personalized therapeutic strategies. It also effectively identifies and discusses key mechanisms, including the NLRP3 inflammasome and leukotrienes, while discussing emerging therapeutic approaches, including neuromodulation and anti-inflammatory treatments, and suggests future research directions. However, several limitations should be considered: the generalizability of the results may be limited by the fact that only eleven studies were ultimately included, and the exclusion of non-English articles and systematic review articles may have excluded relevant studies. This review does not assess the quality or risk of bias of the included studies, which affect the reliability of the results. This may introduce methodological and study design heterogeneity, which complicates the synthesis of evidence. The emphasis on specific biomarkers may result in the neglect of other markers of interest or mechanisms underlying the process. In general, it focuses on short-term outcomes. Therefore, the results may be absent in the long-term rehabilitation process.

## 4. Conclusions

This systematic review underlines neuroinflammation as the hallmark of TBI pathophysiology, negatively affecting neuroplasticity and recovery. Further mechanisms promoting chronic inflammation and impeding repair and recovery of the brain include NLRP3 inflammasomes and leukotrienes, which warrant intervention for anti-inflammation. Microglial activity management may have the potential to improve cognitive outcomes, while SAA1 and TLR4 biomarkers can be used for injury assessment and outcome prediction. Future studies should target specific pathways of neuroinflammation for the development of new therapies, investigate the combination of neuromodulation techniques with anti-inflammatory treatments, and validate biomarkers for clinical application. Studies of neuromodulation techniques such as TMS and tDCS might provide information on their role in inflammation reduction and neuroplasticity improvement. Biomarker profiles may allow for personalized treatments that could offer a better outcome in terms of recovery, and longitudinal studies would be needed to know the long-term effects following such combined treatments.

## Figures and Tables

**Table 1:** Summary of studies included in the research.

Author	Aim	Study Design/Intervention	Treatment Period	Sample Size	Outcomes Measures	Main Findings
Rodriguez et al., 2022 [89]	To study how the NLRP3 inflammasome contributes to neuroinflammation after TBI and its impact on transcriptional and behavioral reactions.	Experimental study.	The pharmacological approach included giving MCC950 (3 mg/kg) before the injury and again 1 h after the injury.	Rats with TBI (number not specified).	The research assessed gene expression patterns, reactions to stimuli, inflammasome constituents, markers of microglia and astrocytes, levels of cytokines, and maintenance of the blood–brain barrier. NSS.	Wild-type mice showed a notable inflammatory reaction following TBI, whereas NLRP3 knockout mice displayed heightened cytokine expression because of increased levels of microglial and astrocyte indicators. MCC950 treatment produced similar outcomes as in mice lacking the gene and enhanced repair when given after the injury.
Jensen et al., 2014 [90]	To assess how effective MK-886, a FLAP inhibitor, is in decreasing the production of leukotrienes, secondary brain damage, synaptic dysfunction, and cognitive impairments after TBI.	Experimental study.	MK-886 was given before and after the injury to assess its ability to prevent and treat the blocking of leukotriene production.	Rats with TBI (number not specified).	Leukotriene levels were assessed with liquid chromatography linked to tandem mass spectrometry; brain swelling was examined with T2-weighted MRI.	MK-886 effectively inhibited leukotriene formation, decreased swelling in the brain, maintained the integrity of the blood–brain barrier in the hippocampal CA1 area, and enhanced synaptic function and cognitive abilities after TBI.
Carabias et al., 2020 [96]	The research sought to establish connections between certain serum biomarkers (S100β, GFAP, NSE, tau, pNF-H, SAA1, CRP, PCT, YKL-40) and the seriousness, size, and site of hemorrhagic TBI lesions.	Prospective observational cohort study.	Not specified.	115 human patients with TBI.	GCS; serum level biomarkers.	SAA1, YKL-40, PCT, and S100β showed a strong correlation with the severity of TBI (based on GCS scores) and the extent of various types of intracranial bleeding. The accuracy in detecting intracranial bleeding was enhanced by combining biomarkers.
Bray et al., 2022 [91]	To determine if replacing trauma-related microglia after TBI can decrease long-term inflammation, enhance cognitive and behavior improvement, and lessen immune response to stimuli like LPS.	Preclinical experimental study.	Microglial replacement was initiated 7 days after the injury, with evaluations being carried out at 30 days after the injury.	Mice (number not specified)	Gene expression in the cortex, complexity of dendrites, amount of myelin, connectivity of neurons, cognitive abilities, reactivity of the immune system, and behavior were assessed.	Mandatory replacement of microglia reversed 90% of gene changes caused by TBI, reduced deficits in neuronal connections, and enhanced cognitive and behavioral results.
Dall’Acqua et al., 2017 [97]	To evaluate the interactions between group and time regarding gray matter changes in healthy individuals and mTBI patients from the early to late phase, and to link these results to cognitive differences, differentiating between GO and PO recovery results.	Longitudinal cohort study.	The assessment period ranged from one week to one year after the injury occurred.	49 mTBI human patients and 49 healthy controls.	Alterations in cortical thickness occur in various brain regions, especially in the prefrontal cortex, along with changes in cognitive performance over time.	Differences in cortical thickness in the prefrontal cortex varied among the groups. Cortical thickening was observed in patients with mTBI, whereas healthy controls displayed typical developmental thinning. In the GO group, cognitive improvement was connected to slight increases in cortical thickness, while the PO group showed an excessive thickening associated with cognitive deterioration.
Aungst et al., 2014 [92]	To examine the histological, neurophysiological, and cognitive impacts of single or repeated mTBI in rats, with emphasis on neuronal loss, synaptic activity, and inflammation.	Experimental study.	Rats were monitored and assessed for 28 days after the injury occurred.	Rats (number not specified).	Examination of neuronal cell death and microglial activation through histology, analysis of synaptic plasticity using LTP in hippocampal slices, and cognitive function assessments including Morris water maze and novel object recognition tests.	Multiple occurrences of mTBI led to notable nerve cell depletion, heightened microglial activity in the hippocampus, hindered LTP, and decreased NMDA receptor reactions. Memory and recognition tasks revealed cognitive deficits. A solitary mTBI also caused alterations in LTP, albeit with less serious consequences in contrast to multiple mTBI.
Carabias et al., 2020 [98]	To find new peptide biomarkers through mass spectrometry and determine if SAA1 indicates the extent of intracranial and extracranial damage in TBI patients.	Prospective observational study.	Serum samples were taken when the patient was admitted, and outcomes were evaluated at hospital discharge and again at 6 months.	120 human patients with TBI.	Volume of lesion inside the skull (determined through CT scan), levels of GOS, and concentrations of biomarkers in the blood.	Levels of SAA1 were strongly linked to the severity of both intracranial and extracranial injuries, were related to other injury indicators, and were a predictor of negative outcomes and death. SAA1 showed strong predictive accuracy with AUC values of 0.90 at discharge and 0.89 at 6 months.
Alins et al., 2021 [99]	Studying how the SAA1-TLR4 axis affects inflammation and outcomes in individuals with TBI and evaluating the potential of using SAA1-TLR4 as a biomarker and target for treatment.	Experimental study.	Not specified.	Vitro and patients with TBI.	Serum concentrations of SAA1, TLR4 mRNA levels in white blood cells, neurobehavioral outcomes, and blood–brain barrier integrity. NSS.	Serum levels of SAA1 demonstrated a direct relationship with the extent of TBI and the results after 6 months. SAA1 showed a correlation with TLR4 mRNA levels as well.
Tapp et al., 2022 [93]	To examine the impact of mechanical SF post-TBI on HPA-axis dysfunction, neuroinflammation, and recovery.	Experimental study.	Mice were subjected to sleep interruption for a period of either 7 or 30 days after the injury occurred.	Mice (number not specified).	The cortical levels of stress-related genes, neuronal activation in the hippocampus and hypothalamic paraventricular nucleus, increase in microglial cells, activation of pro-inflammatory glial signaling genes, electrophysiological assessments, and learning of trace fear conditioning.	Post-TBI SF worsens neuroinflammation, affects HPA-axis response, and hampers hippocampal performance. It enhances microgliosis, changes gene expression associated with stress, and hinders cognitive function.
Zhang et al., 2023 [94]	To study how microRNAs in MSC-derived sEVs can support neurological healing, decrease neuroinflammation, and improve neurovascular restructuring in TBI rats.	Experimental study.	SEVs were given through an IV one day after the injury, with assessments carried out for five weeks after the injury and spatial learning and memory examined between days 31 and 35 after the injury.	Rats (number not specified)	MWM, lesion volume, cell loss, neurovascular remodeling, and neuroinflammation.	Naïve-sEV and vector-sEV therapies enhanced functional improvement, decreased neuronal cell death, suppressed neuroinflammation, and stimulated neurovascular restructuring.
Witcher et al., 2021 [95]	To assess the role of microglia in neuropathology and neuroinflammatory processes at various time points after TBI.	Experimental study.	Time markers of 1 day, 7 days, and 30 days after injury indicate the acute, subacute, and chronic phases correspondingly.	Mice (number not specified)	Gene expression was associated with inflammation, interferon signaling, and neuropathology, as well as cortical dendritic complexity, neuronal connectivity, and cognitive function.	Microglial elimination reversed alterations in gene expression linked to inflammation and neuropathology caused by TBI at 7 and 30 days post-injury. Depletion prevented reductions in neuronal dendritic complexity and connectivity, as well as cognitive impairments caused by TBI.

Legend: traumatic brain injury (TBI), Neurological Severity Scores (NSS), messenger RNA (mRNA), 5-lipoxygenase activating protein (FLAP), magnetic resonance imaging (MRI), long-term potentiation (LTP), Glasgow Coma Scale (GCS), lipopolysaccharide (LPS), mild traumatic brain injury (mTBI), good (GO), poor (PO), serum amyloid A1 (SAA1), computer tomography (CT), Glasgow Outcome Scale (GOS), area under the curve (AUC), serum amyloid A1 protein-Toll-like receptor 4 (SAA1-TLR4), sleep fragmentation (SF), hypothalamic–pituitary–adrenal (HPA), mesenchymal stem/stromal cells (MSC), small extracellular vesicles (sEVs), Morris water maze (MWM).

## Data Availability

Not applicable.
